# A feasibility study on bedside upper airway ultrasonography compared to waveform capnography for verifying endotracheal tube location after intubation

**DOI:** 10.1186/2036-7902-5-7

**Published:** 2013-07-04

**Authors:** Osman Adi, Tan Wan Chuan, Manikam Rishya

**Affiliations:** 1 Department of Trauma and Emergency Medicine, Raja Permaisuri Bainun Hospital, Ipoh, Perak 30990, Malaysia; 2 Department of Trauma and Emergency Medicine, Faculty of Medicine, University of Malaya, Kuala Lumpur 50603, Malaysia

**Keywords:** Bedside upper airway ultrasonography, Endotracheal intubation, Verification, Waveform capnography

## Abstract

**Background:**

In emergency settings, verification of endotracheal tube (ETT) location is important for critically ill patients. Ignorance of oesophageal intubation can be disastrous. Many methods are used for verification of the endotracheal tube location; none are ideal. Quantitative waveform capnography is considered the standard of care for this purpose but is not always available and is expensive. Therefore, this feasibility study is conducted to compare a cheaper alternative, bedside upper airway ultrasonography to waveform capnography, for verification of endotracheal tube location after intubation.

**Methods:**

This was a prospective, single-centre, observational study, conducted at the HRPB, Ipoh. It included patients who were intubated in the emergency department from 28 March 2012 to 17 August 2012. A waiver of consent had been obtained from the Medical Research Ethics Committee. Bedside upper airway ultrasonography was performed after intubation and compared to waveform capnography. Specificity, sensitivity, positive and negative predictive value and likelihood ratio are calculated.

**Results:**

A sample of 107 patients were analysed, and 6 (5.6%) had oesophageal intubations. The overall accuracy of bedside upper airway ultrasonography was 98.1% (95% confidence interval (CI) 93.0% to 100.0%). The kappa value (*Κ*) was 0.85, indicating a very good agreement between the bedside upper airway ultrasonography and waveform capnography. Thus, bedside upper airway ultrasonography is in concordance with waveform capnography. The sensitivity, specificity, positive predictive value and negative predictive value of bedside upper airway ultrasonography were 98.0% (95% CI 93.0% to 99.8%), 100% (95% CI 54.1% to 100.0%), 100% (95% CI 96.3% to 100.0%) and 75.0% (95% CI 34.9% to 96.8%). The likelihood ratio of a positive test is infinite and the likelihood ratio of a negative test is 0.0198 (95% CI 0.005 to 0.0781). The mean confirmation time by ultrasound is 16.4 s. No adverse effects were recorded.

**Conclusions:**

Our study shows that ultrasonography can replace waveform capnography in confirming ETT placement in centres without capnography. This can reduce incidence of unrecognised oesophageal intubation and prevent morbidity and mortality.

**Trial registration:**

National Medical Research Register NMRR11100810230.

## Background

Airway skills are crucial for emergency physicians. In emergency settings, verification of endotracheal tube (ETT) location is important for critically ill patients. Ignorance of oesophageal intubation can be disastrous [1]. This usually happens during intubation in emergency conditions. The incidence of oesophageal intubation was reported at 6% to 16% in emergency conditions [2,3]. Thus, emergency airway efforts in the emergency department (ED) must concentrate on early detection of unintentional oesophageal intubation.

Over the decades, many methods had been used to verify endotracheal intubation. For example, Vaghadia et al. concluded that end tidal carbon dioxide (ETCO2) is most appropriate for identifying oesophageal intubation [4]. Capnography had also been found to be the best method for rapid assessment of tube position [5]. Subsequently, there are reports that the oesophageal detector device has greater accuracy in emergency settings [6]. Although many techniques have been recommended to verify the ETT location, there is no single verification method that is ideal in every situation [7,8]. Nevertheless, there are two studies that are nearest to the ideal. They are two studies of waveform capnography to confirm ETT position in victims of cardiovascular arrest post-intubation which showed 100% sensitivity and 100% specificity in verifying the correct tracheal tube location [9,10]. Therefore waveform capnography is considered the standard of care for the primary verification of endotracheal tube location.

Unfortunately, capnography is not always or freely available, especially in small centres. This is in contrast to ultrasound which is relatively more freely available even in small centres as it is an indispensable tool for managing obstetrics and gynaecology cases and also trauma cases.

Ultrasonography (US) is a widely accessible tool in ED. It is easy to carry, has wide availability, does not cause pain, is relatively cheaper, is easily reproducible and has good safety records [11]. Several studies of ultrasonography confirmation of ETT position provided promising results in a cadaver model [7] or patient in a controlled operating room setting [12,13].

### Literature review

There is a prospective study conducted in Taiwan with the objective of determining the accuracy in diagnosis and timeliness of a novel approach, named tracheal rapid ultrasound exam (TRUE), (which uses convex transducer as opposed to linear transducer, as used in our study) to verify endotracheal tube location during intubation in emergency settings. Quantitative waveform capnography was used as the gold standard for verification of tube placement. A good agreement and concordance between the two aforementioned methods were the outcome. Therefore, it is concluded that the use of TRUE to determine endotracheal tube location during intubation is possible and can be performed quickly [14].

There is a prospective clinical trial in Pusan National University Hospital, South Korea where combined ultrasonography methods using trans-cricothyroid membrane ultrasonography and ultrasonography lung-sliding assessment were found to have 100% sensitivity and specificity in verification of tube placement in emergency settings [15].

A prospective study to compare diaphragmatic ultrasound and chest radiography to verify endotracheal tube location in paediatric emergency settings is done in Cincinnati, USA where they found no equivalence in diaphragmatic ultrasound compared to CXR for tube location in the airway. Nevertheless, ultrasound results were faster, identified more wrong placements and were repeatable among sonographers. Ultrasound results are 8 min faster to obtain than chest X-ray results [16].

In another ultrasound method to verify endotracheal tube placement, a study is done in California, USA. The objective is to calculate specificity and sensitivity of trans-cricothyroid sonography to verify endotracheal intubation. It is a prospective, randomised double-blind trial done in a human cadaver. It was found that dynamic assessment had higher sensitivity and specificity than static assessment. Nevertheless, more tests in living beings are needed to validate these data [7].

In Cleveland, USA, a prospective, randomised, controlled study is done to calculate the accuracy of ultrasonography for identifying the endotracheal tube location in real time. Endotracheal tubes were deliberately placed (with direct laryngoscopy) at random in the trachea and oesophagus. Location of the endotracheal tube was then recorded independently by two blinded physicians. They achieved 100% sensitivity and specificity. Thus, it is concluded that the location of endotracheal tubes during the process of intubation can be accurately identified with ultrasonography in selected patients in controlled settings of the operating room [17].

Galicinao and colleagues had done a study to assess the use of ultrasound in verifying ETT location among paediatric patients. It showed that linear transducer provides better images but is limited by its size compared to curvilinear ones and has better timeliness of ultrasound as compared to chest radiograph; sniffing is the best position for high-quality images. They also showed that ultrasound can be used accurately when ETCO2 detector shows wrong or ambiguous results [18].

There is a recent prospective pilot study of newborns admitted to the San Diego Medical Centre that found a good correlation between ultrasound and radiograph in determining the anatomical position of ETT in preterm and term infants [19]. In Augusta, USA, ultrasound capture of the lung-sliding sign in a cadaver had been found to be accurate in detecting ETT placement in the trachea, oesophagus and right main stem bronchus.

In a paediatric intensive care unit, Hsieh et al. used the motion of the diaphragm to determine the position of ETT placement. It is thus recommended for the secondary confirmation of the ETT position [20].

In pre-hospital or disaster settings, Chun et al. used handheld ultrasound to confirm ETT placement in extreme conditions where auscultation and capnography may not be appropriate. Their report suggests that thoracic ultrasonography could be used to verify proper ETT placement [21].

### The research question

Is bedside upper airway ultrasonography in good agreement with capnography in verifying endotracheal tube placement after intubation?

### Objectives

The following are the objectives of the study:

1. General

(a) To assess the feasibility of bedside upper airway ultrasonography verification of endotracheal intubation as compared to waveform capnography, which is the standard of care in primary/immediate verification process

2. Specific

(a) To verify ETT location in patients in the ED with bedside upper airway ultrasonography and to compare it with waveform capnography (gold standard)

(a) To estimate verification time by ultrasound

3. Hypothesis

(a) Bedside ultrasonography is in good agreement with the ‘gold standard’ test, waveform capnography.

(a) The mean verification time is about the same as other tracheal ultrasound techniques by other investigators, which is less than 30 s.

### Ethical consideration

There is a theoretical possibility of the mentioned investigations causing delay in sending patient to the ward. However, firstly, we would like to clarify that US confirmation of endotracheal intubation takes only a mean time of 14 s, and the maximum time between capnography and US is 60 s [14]. Thus, it seems that the little delay looks negligible. Secondly, all clinical decisions are dependent on capnography results, and ultrasonography will not be involved in clinical decision-making. All patients will receive routine treatment, and ultrasound would be the only additional investigation added and will not interfere in patient care. Thirdly, previous researches on ultrasound confirmation had a waiver of consent due to the nature of working environment in the emergency department.

Physicians have used ultrasound for many decades. Until now, researchers have not found any side effects clearly caused by ultrasound. Each year, millions of babies born had undergone ultrasound scanning *in utero*. This is an enviable safety record. Hence, ultrasonography is not harmful to the patients. It is generally viewed as a safe imaging modality [22]. The World Health Organization technical report series (1998) states that ultrasound is generally harmless [23]. A meta-analysis of several published studies (year 2000) reported that ultrasonography had no statistically significant side effects [24].

Ultrasound is an indispensable tool for emergency physicians to quickly diagnose salvageable conditions in critically ill patients. This is because the usual methods to diagnose will be cumbersome, risky and not timely enough for unstable patients where time is an important factor in determining survivability of the patients. This application is called point-of-care ultrasound [25]. This research falls into this category. Since US confirmation of endotracheal intubation takes only a mean time of 14 s [14], any hypothetical risks to the patients seem negligible. There are no known harmful patient outcomes with the use of sonography in the ED in a study done in the UK [26]. Furthermore, legal actions have been taken against physicians for not performing point-of-care ultrasound [27].

Fourthly, obtaining informed consent from the patients is impossible because all intubated patients are unconscious, ill and often unstable. Furthermore, their relatives are often not around. Even if they are around, consent will cause significant delay to the care of the patient. This will defeat the whole purpose of emergency ultrasound, which is meant to be kept simple and as fast as possible [28]. The ultrasound in this research takes only a mean time of 14 s [14]. To take consent will take minutes to hours which will cause unacceptable delays in patient care. There are no other ways to obtain the information of this research without the patient being unconscious (this research is about intubated patients in the emergency department, and all intubated patients here are unconscious). We think that the potential benefit to the population as a whole outweighs the individual right of the study patients to absolute identification. The reason for this research is important; it is to show that ultrasound is in good agreement to waveform capnography which is the gold standard in the immediate confirmation of endotracheal intubation. Not all hospitals have the facilities of ETCO2, but all district hospitals at least have US at their disposal. If this is successful, we could train all district MOs to use US to confirm ETT placement and thus reduce incidences of unrecognised oesophageal intubation which are sometimes difficult to detect clinically but may cause substantial morbidity and mortality. Medical Research Ethics Committee of Malaysia had given a waiver of consent. This study is registered with the National Medical Research Register and approved by the Clinical Research Centre and National Institute of Health of Malaysia.

### Rationale of study

The reason for this research is important; it is to show that bedside upper airway ultrasound is in good agreement to waveform capnography which is the gold standard in the primary/immediate verification of endotracheal intubation. Primary/immediate verification means that verification done before endotracheal tube is secured. Not all hospitals have the facilities for capnography, but they at least have US at their disposal. If this is successful, ultrasound can be used in centres without capnography to reduce incidences of unrecognised oesophageal intubation which are sometimes difficult to detect clinically but may cause substantial morbidity and mortality.

## Methods

### Research methodology

The following is the research methodology used:

1. Study design: it is a prospective, single-centre and observational study.

2. Population and sample:

(a) Reference population: all patients intubated in the emergency department of Raja Permaisuri Bainun Hospital, Ipoh, Perak, Malaysia, (which is a 990-bed, tertiary hospital), whenever the investigators were available.

(a) Source population: all patients that are intubated by an emergency department medical officer or physician in the red zone in the emergency department of Raja Permaisuri Bainun Hospital.

3. Criteria:

(a) Inclusion criteria:

• Patients who are included are those requiring intubation in the emergency department because of respiratory failure, comatose, cardiac arrest and others.

• Only first attempt of intubation is included.

(a) Exclusion criteria:

• Patients who are excluded are those with anatomical neck distortion of any cause. This is because distorted anatomy of the neck makes ultrasonography interpretation impossible.

4. Study sample: all intubated patients in the emergency department who fulfils the inclusion and exclusion criteria.

5. Sampling method: all patients that are intubated in the emergency department HRPB will be used in the study. They were enrolled whenever the investigators were available.

6. Sample size: the sample size was 107 for the primary outcome. It is estimated from the expected sensitivity of 0.99, expected specificity of 0.94, prevalence of oesophageal intubation as 0.15, with desired precision of 0.05 and confidence level of 95% based on research done by Chou H-C [14]. This is based on a simple nomogram for sample size for estimating sensitivity and specificity of medical tests [29].

7. Detailed description:

(a) Intubation was done by emergency residents.

(b) Meanwhile, the researcher performs upper airway ultrasonography examinations for endotracheal tube placement.

(c) Then endotracheal tube placement will then be confirmed by quantitative waveform capnography.

(d) Endotracheal intubation confirmation is interpreted when exhaled CO2, which is ≥4 mm Hg after more than or equal to five breaths, is detected with a characteristic CO2 waveform.

(e) The endotracheal tube is secured only after its placement in the trachea is confirmed by quantitative waveform capnography.

(f) The ultrasonography technique used is bedside upper airway ultrasonography. We placed a linear ultrasonography transducer over the whole of the upper airway from upwards to downwards until we found the ETT image. We then took the image of the ETT in transverse and longitudinal views. The probe is then moved to the left to look at the oesophagus to see whether it is empty or distended by ETT.

(g) Upon completion of the study, the result of bedside ultrasonography will be compared with quantitative waveform capnography. Sensitivity, specificity and positive predictive value of bedside ultrasonography examination are computed to determine the accuracy and effectiveness of clinical use. Using kappa statistics, we will use the values to determine the strength of agreement between bedside ultrasonography examination and waveform capnography as the standard of care.

(h) The verification time required by ultrasound is recorded. The verification time is defined as after endotracheal tube insertion had been completed to the ultrasound verification results. The mean and standard deviation of the confirmation time using ultrasonography will be calculated using STATA software version 12.0.

#### Operational definitions

**Figure 1:**
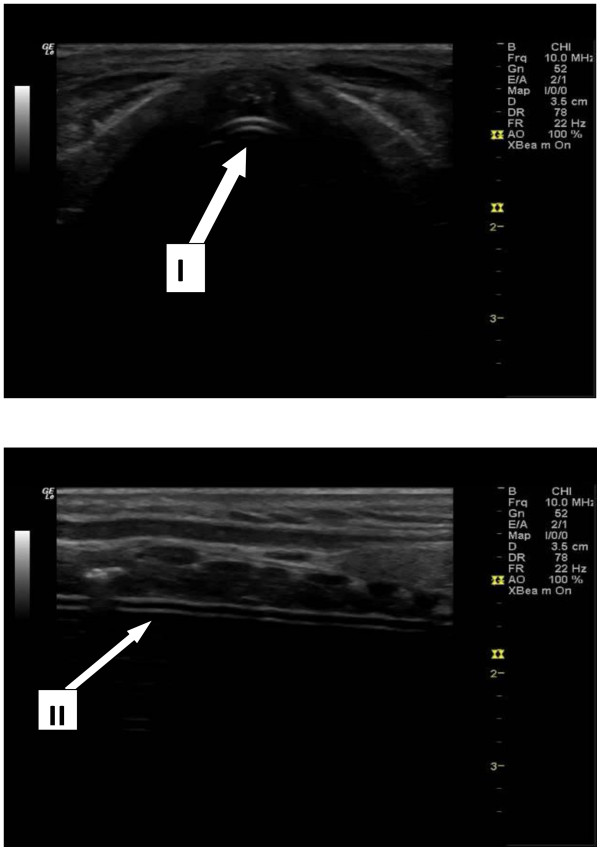
**Tracheal intubation.** The images show endotracheal intubation (ETT) in correct position, as seen in the horizontal view (I); the arrow indicates two hyper-echoic lines, which means the ETT is inside the trachea. On the vertical view (II), two symmetrical hyper-echoic lines indicate the ETT.

A LOGIQ e series ultrasound scanner by GE Medical Systems Co. Ltd. (Little Chalfont, UK) equipped with a 10 MHz linear transducer was used for sonography. After completion of intubation, the endotracheal tube location is instantly verified. The probe is orientated horizontally on the whole upper airway from cricothyroid membrane to the suprasternal notch. In the horizontal and vertical view, two symmetrical hyper-echoic lines indicate that the ETT is inside the trachea (Figure 1). The oesophagus will be viewed as empty if the ETT is inside the trachea.

**Figure 2:**
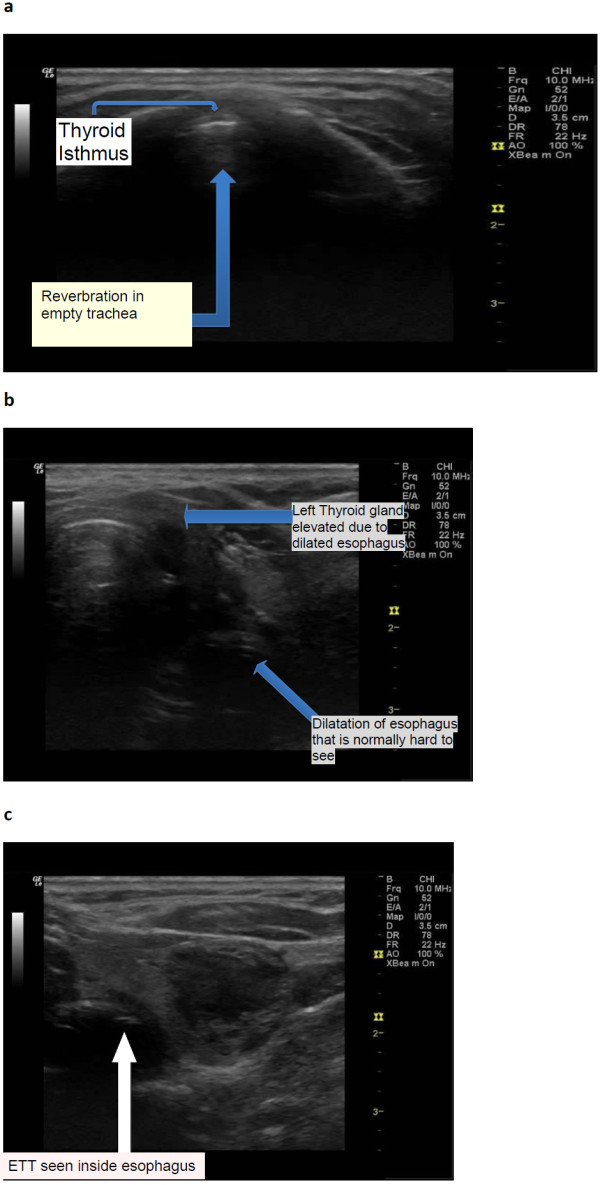
**Oesophageal intubation. (a)** US shows empty trachea. **(b)** Moving the probe to the left shows a dilated oesophagus. **c**. Focus on the oesophagus shows an endotracheal tube inside the oesophagus evidenced by two hyper-echoic lines inside oesophagus.

The verified endotracheal tube location was interpreted as (1) the tube inside the trachea if in horizontal view; the presence of the two hyper-echoic parallel lines confirms the presence of ETT, or in vertical view, two hyper-echoic parallel lines indicate the ETT. (2) It is oesophageal intubation if there is the absence of two hyper-echoic parallel lines in both horizontal and in vertical views which signify an empty trachea plus distended oesophagus with the presence of two hyper-echoic parallel lines which signify ETT in the oesophagus (Figure 2).

#### Study flow diagram

**Figure 3:**
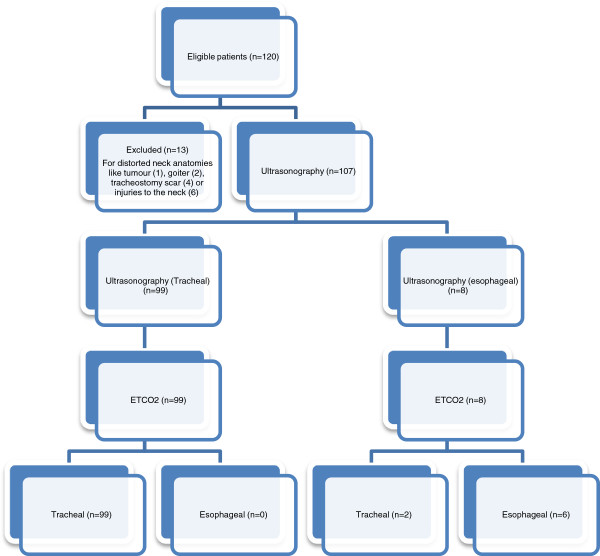
Study flow diagram.

A study flow diagram is shown in Figure 3.

#### Research tools

The following are the research tools used in the study:

**Figure 4:**
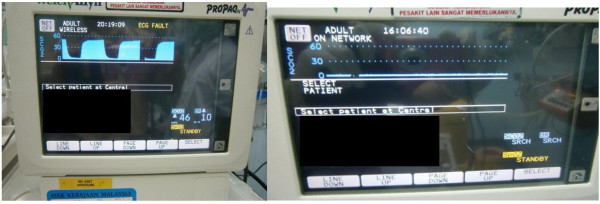
**Quantitative waveform capnography (ETCO2).** The picture on the left shows a positive quantitative waveform capnography (ETCO2), which means ETT is in the trachea (correct placement). The picture on the right shows a negative ETCO2, which means ETT is not inside the trachea.

▣ The emergency medical officers will perform the intubation.

▣ The proposed ultrasonography verification of endotracheal intubation was done by a resident and physician of emergency medicine who had successfully undergone training in ultrasonography.

▣ The resident (investigators) had been trained on airway ultrasonography by WINFOCUS Ultrasonography Life Support (WinFocus Malaysia Group, Kuala Lumpur, Malaysia). He was under the supervision of an emergency medicine physician.

▣ The patients' histories and capnography were not known to the ultrasonographers.

▣ Post-intubation verification of endotracheal tube location was validated by an emergency medicine resident by clinical signs, quantitative waveform capnography (Figure 4) and also pulse oximetry.

▣ All clinical decisions are dependent on capnography results, and ultrasonography will not be involved in clinical decision-making.

#### Outcome measures

The primary outcome is the strength of agreement between bedside ultrasonography and capnography in confirming endotracheal tube location after intubation. The secondary outcome is the total time taken to complete the ultrasound procedure.

#### Data collection and processing

All enrolled subjects' data such as gender, age, race, indications of intubation, ultrasonographic image and results, and sonographic verification time were collected on a data entry sheet. An ultrasound machine was used to record each of the images. Ultrasonographers were to jot down the location of the endotracheal tube and save an image of each subject.

Kappa values will be calculated from Table 1. Kappa statistic is used because it gives the strength of agreement over and above that which would have occurred just by chance.

**Table 1:** Ultrasonography confirmation of tracheal and oesophageal intubation

**Ultrasound**	**Quantitative waveform capnography**		
	**Endotracheal intubation**	**Oesophageal intubation**	**Total**
Endotracheal intubation	99	0	99
Oesophageal intubation	2	6	8
Total	101	6	107

It will be interpreted based on the following criteria [30]:

•Kappa values from 0.81 to 1.00 are very good.

•Kappa values from 0.61 to 0.80 are good.

•Kappa values from 0.41 to 0.60 are moderate.

•Kappa values from 0.21 to 0.40 are fair.

•Kappa values <0.20 are poor.

We will then calculate the specificity, sensitivity and positive and negative predictive values as well as likelihood ratio.

#### Data entry

Data is entered and analysed using STATA version 12.0. The data entry sheet could be viewed in Additional file 1.

## Results and discussion

### Results

A sample of 107 patients were analysed, and 6 (5.6%) had oesophageal intubations. The overall accuracy of bedside upper airway ultrasonography was 98.1% (95% confidence interval (CI) 93.0% to 100.0%). The kappa value (*Κ*) was 0.85, indicating a very good agreement between the bedside upper airway ultrasonography and waveform capnography. Thus, bedside upper airway ultrasonography is in concordance with waveform capnography. The sensitivity, specificity, positive predictive value and negative predictive value of the bedside upper airway ultrasonography were 98.0% (95% CI 93.0% to 99.8%), 100% (95% CI 54.1% to 100.0%), 100% (95% CI 96.3% to 100.0%) and 75.0% (95% CI 34.9% to 96.8%). The likelihood ratio of a positive test is infinite, and the likelihood ratio of a negative test is 0.0198 (95% CI 0.005 to 0.0781). The mean confirmation time by ultrasound is 16.4 s. No adverse effects were recorded while performing bedside upper airway ultrasonography (Tables 1, 2 and 3; Additional files 2 and 3).

**Table 2:** Bedside upper airway sonography characteristics for intubation confirmation

	**Value**	**95% Confidence interval**	
		**Lower limit**	**Upper limit**
Sensitivity, %	98%	93%	99.8%
Specificity, %	100%	54.1%	100%
Positive predictive value	100%	96.3%	100%
Negative predictive value	75%	34.9%	96.8%
Likelihood ratio (+)	Infinity	-	-
Likelihood ration (−)	0.0198	0.005	0.0781

**Table 3:** Confirmation time for endotracheal tube placement

	**Total (s)**
Mean ± SD	16.4 ± 7.33
Median	14
IQR	12.0, 18.0

SD, standard deviation; IQR, interquartile range.

### Discussion

The structure of discussion in the next subsections is based on the work of Docherty and Smith [31].

#### Principal findings

This research shows a very good agreement, with a kappa value of 0.85, between bedside upper airway ultrasound and waveform capnography and also a quick mean confirmation time of 16.4 s with a standard deviation of 7.3 s. Therefore, this study suggests that bedside upper airway ultrasonography can be used in the primary confirmation of endotracheal tube placement in centres without waveform capnography. This technique was time-saving and manifold faster than chest radiographs [18].

#### Strength of the study

This study covers a great variation of patients who differ significantly in age, ethnic group and indications of intubation. This also represents an opportunity for the generation of a protocol for the use of bedside upper airway sonography in the primary verification of endotracheal tube location. Unlike previous studies which only scanned parts of the upper airway, this study involves scanning the whole upper airway and the oesophagus. The scanning of the oesophagus proved to be invaluable to exclude oesophageal intubation.

Upper airway ultrasonography can also be advantageous in situations involving cardiovascular arrest, bronchoconstrictions or others in which capnography or ETCO2 might be faulty [32-34]. As recommended by Chun and colleagues [21], there is enormous potential in the use of ultrasonography in extreme environments like aeromedicine and disasters where usual equipment to verify ETT positions is difficult, if not impossible.

This requires adequate training and experience, which is accessible to most medical providers. Thus, it is evident that bedside upper airway ultrasonography should be the method of choice in the primary verification of endotracheal tube location in the upper airway.

#### Limitations of the study

Nevertheless, this study had some limitations. There might be subtle biases which can be missed because of eventual successful endotracheal intubations for each patient. Furthermore, application of ultrasonography for general use needs to be determined owing to the small number of investigators involved in the single-centre study. The sample size of 107 might not be big enough to represent the whole population.

In this research, bedside upper airway ultrasonography misidentified two cases (two false negatives). False negative is defined as tracheal intubation falsely determined as oesophageal intubation. They should be closely examined and potential reasons should be explained. This is because the accuracy of static assessments was possibly less than that of the dynamic ones. In those cases, after ultrasonography verification had been completed, the patients were subsequently found out to have subcutaneous emphysema due to pneumothorax which makes the identification of the two hyper-echoic lines difficult and therefore leading the investigator to conclude oesophageal intubation. Those images also had poor quality.

Moreover, we did not sufficiently investigate oesophageal intubations. The rate for oesophageal intubation during the initial intubation was 5.6% (6 out of 107 cases) with only six oesophageal intubation noted as compared to that previously reported by Schwartz [2]. This may cause the study's high specificity and sensitivity. Thus, the bias is because every intubation was done under the supervision of a senior medical officer or emergency physician. Patients who were intubated outside the emergency department, for example, patients who were from extra-hospitals settings, are excluded from this study as confirmation time could not be calculated. Usually, patients who were intubated in extra-hospitals settings were done by less experienced personnel. A future study has to be performed in centres where the success rate is low, such as non-hospital settings.

#### Comparison with other studies

There are a few methods of ultrasound that had been used to confirm ETT placement. It can be divided into direct and indirect methods. The direct methods involve viewing the trachea as employed by this study. It is different from TRUE [14] and trans-cricothyroid membrane ultrasonography [7]. TRUE utilises a convex transducer in the suprasternal notch window, whereas transcricothyroid membrane ultrasound uses a linear transducer on the cricothyroid membrane as the name implies. However, a convex transducer has a lower frequency which makes visualisation of superficial structures difficult, and anatomical artefacts such as thyroid gland calcification can cause a false negative [14]. Trans-cricothyroid membrane ultrasonography uses the cricothyroid membrane as scanning position and entirely depends on the appearance change of the vocal cords, but a study had shown that only 71% of the healthy human subjects were visible by ultrasound [35]. In the preliminary study of ultrasound of the vocal cords and larynx, real-time ultrasound image of the vocal cords and larynx had been successfully used to guide the endotracheal intubation of an awake, healthy volunteer. However, it had been unsuccessful in anaesthetised patients because of the difficulty in viewing the advancing tip of the endotracheal tube [36]. Our bedside upper airway ultrasonography utilises linear probe and uses the whole trachea as scanning position.

Ultrasound assessment can also be done in a dynamic or static manner. We use the static manner for bedside upper airway ultrasonography. Though the dynamic manner might have better accuracy, it can disturb the process of intubation [7].

The indirect methods of ultrasound confirmation used by previous investigator utilise other two windows like intercostal and sub-xiphoid windows or diaphragm [37]. They are based on detecting the pleura's sliding or diaphragm movement [20,38]. Nevertheless, these methods involve positive pressure ventilation and can be influenced by underlying lung pathology like pneumothorax, pneumonia etc.

#### Meaning of the study

After intubation, there are primary and secondary verifications of endotracheal tube [16]. Primary verifications are defined as procedures performed before the endotracheal tube is secured, which includes direct observation of the tube going through the glottis, the rise of the chest, presence of vapour in the tube, auscultation of breath sounds and quantitative waveform capnography measurement.

Bedside upper airway ultrasound is a method that directly observes the upper airway structures in real time to identify endotracheal tube location to determine whether it is in the trachea or in the oesophagus. Therefore, it falls in the primary verification procedures.

Secondary verifications like chest X-rays are used to determine the location of the ETT inside the airway after primary verification has ruled out non-airway locations.

#### Implications for clinicians/policymakers

Ultrasound is becoming very important in upper airway management in emergency care settings [14-17]. This is because it is easy to carry, relatively cheaper, proven to have safety records, freely available, does not cause pain and easily reproducible [11].

This research shows that bedside upper airway ultrasound is in very good agreement with waveform capnography which is the gold standard in immediate verification of endotracheal intubation. Capnography is not as freely available or applied with consistency even among emergency physicians [39]. Ultrasound is always available in the emergency department. All it needs, to enable bedside upper airway ultrasonography, is a personnel trained in upper airway ultrasound and a linear ultrasound probe.

Hence, medical officers in centres without waveform capnography should be trained to use ultrasonography to confirm endotracheal tube placement and thus reduce incidences of unrecognised oesophageal intubation which are sometimes difficult to detect clinically, even by auscultation of the chest [40], but may cause substantial morbidity and mortality.

Although capnography had become a standard of care because it is simple to use and very reliable [41], capnography by itself is inadequate for endotracheal tube location verification after intubation [42]. The reliability of quantitative capnography is a suspect in some low pulmonary flow condition like cardiac arrest [32]. There are suggestions that six breaths were needed to clear the stomach of carbon dioxide, especially after prolonged bag-valve-mask ventilation [43]. Low pulmonary flow will not disturb sonography images. Compared to direct visualisation during laryngoscopy, ultrasound allows verification of endotracheal tube location without disturbing ongoing cardiopulmonary resuscitation and with a decreased risk of unintended extubation [44].

This research also shows that bedside upper airway ultrasonography has a quick mean time of 16 s. Other methods of ultrasonography verification of endotracheal tube placement such as TRUE also shows a fast confirmation time of 14.8 s [14]. Among patients with obesity, verification of endotracheal tube location by sonography is as quick as auscultation alone and quicker than the usual methods of auscultation and capnography [45]. Since US confirmation is quick enough; it can be used to identify oesophageal intubation before pumping air to the patient and prevents unintended stomach ventilation. Therefore, ultrasound can replace capnography in the primary verification of ETT location, in centres with no capnography facility.

#### Unanswered questions and future research

Since bedside upper airway ultrasound imaging depends on the identification of normal upper airway anatomical structures and relationships, the use in patients with goitre, tumours of the neck or injuries could not be investigated. Future studies should concentrate on methods to verify endotracheal tube location in patients with distorted anatomy and also to assess the possibility of teaching bedside upper airway ultrasonography.

## Conclusions

This study indicates that the use of bedside upper airway ultrasonography to verify endotracheal tube location in the primary verification process is feasible and can be easily and quickly done.

## Abbreviations

CO2: Carbon dioxide; ED: Emergency department; ETT: Endotracheal tube; US: Ultrasonography.

## Competing interests

The authors declare that they have no competing interests.

## Authors’ contribution

ABO was involved in study conception, study design, discussion, methodology, completed data acquisition, collection and results interpretation, statistical calculation and analysis, result interpretation, and discussion. TWC completed write-up of the manuscript and was involved in study conception, study design, discussion, methodology, data collection, statistical calculation and analysis, result interpretation, and discussion. RM contributed to the initial conception, study design as well as intellectual contents of the study. All authors read and approved the final manuscript.

## Supplementary Material

Additional file 1 Data entry sheet. Click here for file

Additional file 2 Data entry 1. Click here for file

Additional file 3 Data entry 2. Click here for file
