# Supplementary material for: A feasibility study on bedside upper airway ultrasonography compared to waveform capnography for verifying endotracheal tube location after intubation
Source: Crit Ultrasound J. 2013 Jul 4;5(1):7. doi: 10.1186/2036-7902-5-7 (PMC3772703; doi:10.1186/2036-7902-5-7)
Supplement: Additional file 1 — Data entry sheet. [file 2036-7902-5-7-S1.doc]

**Data entry sheet**

Age : ______________________

Race : Malay ( ) Chinese ( ) Indian ( ) Others ( )

Gender: Male ( ) Female ( )

Intubation indications : respiratory distress ( ) cardiac arrest ( ) comatose ( ) others ( )

Intubation time : ____________s

Ultrasound image: tracheal intubation ( ) esophageal intubation ( )

Quantitative waveform CO2: tracheal intubation ( ) esophageal intubation ( )
