# Supplementary material for: A feasibility study on bedside upper airway ultrasonography compared to waveform capnography for verifying endotracheal tube location after intubation
Source: Crit Ultrasound J. 2013 Jul 4;5(1):7. doi: 10.1186/2036-7902-5-7 (PMC3772703; doi:10.1186/2036-7902-5-7)

**Data entry sheet 2**

Age : ________34_____________

Race : Malay ( / ) Chinese ( ) Indian ( ) Others ( )

Gender : Male ( / ) Female ( )

Intubation indications : respiratory distress ( ) cardiac arrest (/ ) comatose ( ) Others ( )

Confirmation time of tracheal placement : _14___________s

Ultrasound image: tracheal intubation ( / ) esophageal intubation ( )


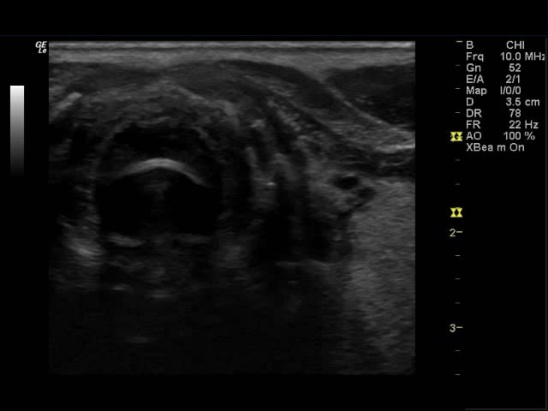

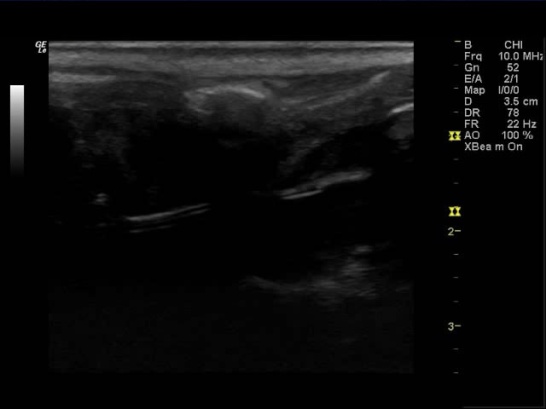


Quantitative waveform CO2: tracheal intubation[positive] ( / ) esophageal intubation[negative] ( )


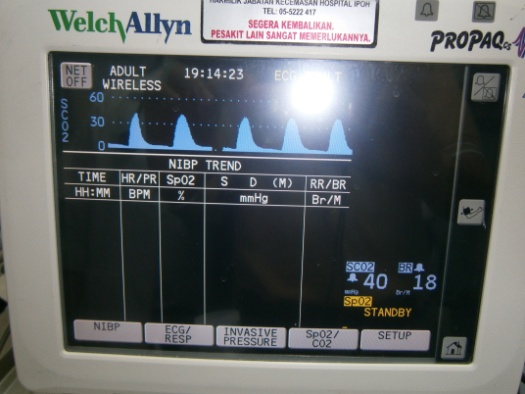

Supplement: Additional file 3 — Data entry 2. [file 2036-7902-5-7-S3.zip › Additional file 3/Data entry 2/tracheal intubation/Data entry sheet 2.docx]
