# Supplementary material for: A feasibility study on bedside upper airway ultrasonography compared to waveform capnography for verifying endotracheal tube location after intubation
Source: Crit Ultrasound J. 2013 Jul 4;5(1):7. doi: 10.1186/2036-7902-5-7 (PMC3772703; doi:10.1186/2036-7902-5-7)

**Data entry sheet 7**

Age : ______20________________

Race : Malay ( ) Chinese ( ) Indian (/ ) Others ( )

Gender : Male ( / ) Female ( )

Intubation indications: respiratory distress ( ) cardiac arrest ( ) comatose ( ) Others ( / )

Confirmation time of tracheal placement : ____16________s

Ultrasound image: tracheal intubation (/ ) esophageal intubation ( )


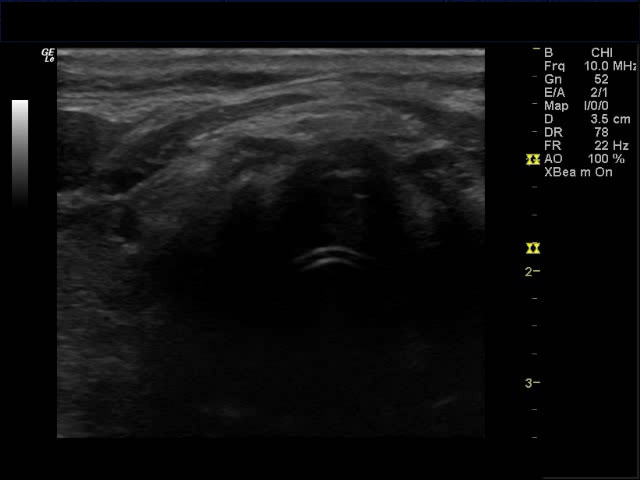

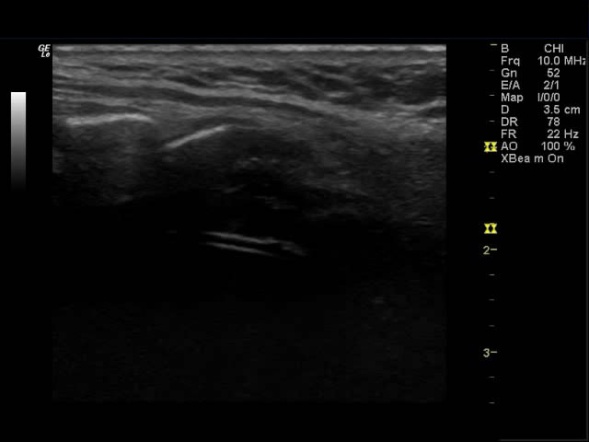


Quantitative waveform CO2: tracheal intubation [positive]( /) esophageal intubation[negative] ( )


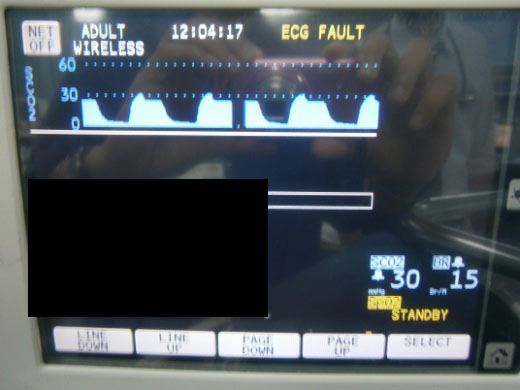

Supplement: Additional file 3 — Data entry 2. [file 2036-7902-5-7-S3.zip › Additional file 3/Data entry 2/tracheal intubation/Data entry sheet 7.docx]
