# Supplementary material for: A feasibility study on bedside upper airway ultrasonography compared to waveform capnography for verifying endotracheal tube location after intubation
Source: Crit Ultrasound J. 2013 Jul 4;5(1):7. doi: 10.1186/2036-7902-5-7 (PMC3772703; doi:10.1186/2036-7902-5-7)
Supplement: Additional file 2 — Data entry 1. [file 2036-7902-5-7-S2.zip › Additional file 2/Data entry 1/esophageal intubation/Data entry sheet 102.doc]

**Data entry sheet 102**

Age	: ___________87___________

Race	: Malay (	)	Chinese (/ )	Indian (	)	Others (	)

Gender : Male	(	)	Female ( / )

Intubation indications	: respiratory distress (	) cardiac arrest (/ ) comatose (	) Others (	)

Confirmation time of tracheal placement	:	___34_________s

Ultrasound image: tracheal intubation ( )	esophageal intubation ( / )

Quantitative waveform CO2: tracheal intubation [positive]( )	esophageal intubation[negative] ( /	)
