# Supplementary material for: A feasibility study on bedside upper airway ultrasonography compared to waveform capnography for verifying endotracheal tube location after intubation
Source: Crit Ultrasound J. 2013 Jul 4;5(1):7. doi: 10.1186/2036-7902-5-7 (PMC3772703; doi:10.1186/2036-7902-5-7)
Supplement: Additional file 2 — Data entry 1. [file 2036-7902-5-7-S2.zip › Additional file 2/Data entry 1/esophageal intubation/Data entry sheet 16.doc]

**Data entry sheet 16**

Age	: _____________66_________

Race	: Malay ( / )	Chinese (	)	Indian (	)	Others (	)

Gender : Male	(	)	Female ( / )

Intubation indications	: respiratory distress (	) cardiac arrest ( / ) comatose (	) Others (	)

Confirmation time of tracheal placement	:	_____15_______s

Ultrasound image: tracheal intubation (	)	esophageal intubation ( /	)

Quantitative waveform CO2: tracheal intubation [positive](	)	esophageal intubation[negative] (	/ )
