# Supplementary material for: A feasibility study on bedside upper airway ultrasonography compared to waveform capnography for verifying endotracheal tube location after intubation
Source: Crit Ultrasound J. 2013 Jul 4;5(1):7. doi: 10.1186/2036-7902-5-7 (PMC3772703; doi:10.1186/2036-7902-5-7)
Supplement: Additional file 3 — Data entry 2. [file 2036-7902-5-7-S3.zip › Additional file 3/Data entry 2/tracheal intubation/Data entry sheet 99.doc]

**Data entry sheet 99**

Age	: _____68_______________

Race	: Malay (	)	Chinese ( / )	Indian (	)	Others (	)

Gender : Male	(/ )	Female ( )

Intubation indications	: respiratory distress ( ) cardiac arrest (/ ) comatose (	) Others (	)

Confirmation time of tracheal placement	:	______9_____s

Ultrasound image: tracheal intubation (/ )	esophageal intubation (	)

Quantitative waveform CO2: tracheal intubation [positive]( / )	esophageal intubation[negative] (	)
